# Study on Betaine and Growth Characteristics of *Lycium chinense* Mill. in Different Cultivation Environments in South Korea

**DOI:** 10.3390/plants13162316

**Published:** 2024-08-20

**Authors:** Hyejung Cho, Dong Hwan Lee, Dae Hui Jeong, Jun Hyuk Jang, Yonghwan Son, Sun-Young Lee, Hyun-Jun Kim

**Affiliations:** 1 Forest Medicinal Resources Research Center, National Institute of Forest Science, Yeongju-si 36040, Republic of Korea; whgpwjd@korea.kr (H.C.); leedh0419@korea.kr (D.H.L.); najdhda@korea.kr (D.H.J.); wnseldu123@korea.kr (J.H.J.); thsdydghks@korea.kr (Y.S.); nararawood@korea.kr (S.-Y.L.); 2 School of Environmental Engineering, University of Seoul, Seoul 02504, Republic of Korea

**Keywords:** correlation analysis, goji berry, *Lycium chinense*, marker compound, method validation, UHPLC-MS/MS

## Abstract

Betaine is a useful compound that has various activities and is the marker compound of *Lycium chinense* fruit in Korean Pharmacopoeia. we seek to support the stable production of medicinal goji berries, which have significant potential in the pharmaceutical industry due to their high values, and to provide foundational data for consistent quality control. This study’s purpose was to examine the correlation among betaine content, environmental variables, and the growth characteristics of *L. chinense* fruits. The fruits were collected from 25 cultivation sites across South Korea. We investigated five growth characteristics and betaine contents in *L. chinense* fruits and twelve soil physicochemical properties, and seven meteorological data at cultivation sites. The fruit’s growth characteristics included a length of 15.62–26.49 mm, a width of 7.09–11.38 mm, a fresh weight of 0.73–1.62 g, and a sugar content of 11.10–19.62 Brix°. Its betaine content ranged from 0.54% to 0.97%. The betaine content was positively correlated with electrical conductivity (0.327 **), exchangeable potassium (0.314 **), and sodium (0.259 *) and negatively correlated with annual average minimum temperature (−0.256 *) and annual average temperature (−0.242 *). Also, betaine showed a positive correlation with the length of the fruit (0.294 *) and the fresh weight of the fruit (0.238 *). These results can be used to find the best cultivation method and to manage quality control for the highly economical *L. chinense* fruit.

## 1. Introduction

*Lycium chinense* Mill. belongs to the Solanaceae family and is a deciduous tree that is native to or cultivated in Korea, China, Japan, and other countries. These trees can grow up to 2 m in length on hillsides, hills, and other similar areas. The fruit of the *L. chinense* is spindle or oval-shaped, measuring 6–20 mm in length and 3–10 mm in diameter [1]. In Korea, the bark of the root is known as “Ji-gol-pi”, the leaves are called “Gu-gi-yeop”, the young shoots are referred to as “Cheong-jeong-cho”, and the fruit is known as “Gu-gi-ja” [2].

Berries contain a wide range of bioactive substances that have been shown to promote human health, and people around the globe enjoy them [3,4]. Berries have earned a reputation as superfoods [5] and functional foods [6] and are widely recognized for their health benefits. The berry of *L. chinense* is known as Wolfberry or Goji berry. This berry is highly valued in traditional herbal medicine, as well as in the food and medical industries, and is gaining popularity worldwide [7]. Goji berry is a precious herb belonging to a group of 120 non-toxic medicinal herbs, including ginseng [2], and has been used as medicine and functional food for more than 2500 years in many Asian countries. Its widespread use and recognition over such a long period of time is a testament to its effectiveness and significance in traditional medicine [8,9,10]. Based on a previous research, *L. chinense* fruit has been found to enhance liver and kidney function, positively influence the immune system, and offer antioxidant and protective benefits to cells when taken for a prolonged period [11]. It has also been shown to be beneficial in reducing reproductive function decline due to aging, improving vision in cases of arthritis and ophthalmic diseases such as waist and knee, and can be used to treat early symptoms of senile cataracts [12].

Goji berry is known to contain components such as betaine, polysaccharides, polyphenols, carotenoids, alkaloids, carbohydrates, and lignans, which may offer numerous benefits [13]. Some countries set quality standards for herbs as legal regulations. For instance, the Korean Pharmacopoeia has set a standard of 0.5% betaine content for Gugija [14], while the Chinese Pharmacopoeia has set standards of 1.8% polysaccharides and 0.3% betaine content [15].

Betaine is a stable, nontoxic natural component in goji berry. It is consumed through dietary intake or synthesized as a metabolism of choline in the body. The betaine shows throughout a methyl-group donor or an organic osmolyte [16]. In several studies when it was taken into the body, betaine reduced the accumulation of damaged proteins and a phenomenon of alcohol set hepatic steatosis. In short, betaine has anti-inflammatory and antioxidant properties and prevents liver damage [17]. Concretely, the main physiological effects of betaine are to positively affect exercise performance, muscle strength, liver function, and physical condition. It protects the body from metabolic-related fatty liver disease, myocardial function, cancer development, and pancreatic steatosis, and also plays a neuroprotective role [18,19].

The content of compounds in plants and their growth are affected by the environment in which they grow. Two research papers reported the influence of various factors on the compounds of goji berry. One paper from China noted that cultivars and micro-environmental conditions such as soil salt, pH, organic matter, and others can impact the fruit’s compounds [20]. The other paper reported that several factors, including the geographic origin, climate, soil type, and harvesting period, can influence the polyphenol components in goji berry fruits from European cultivars [21]. Betaine has been reported to play a role in protecting plants from various abiotic stresses such as high salinity, extreme temperatures, and drought [22]. Therefore, the content of betaine, a marker compound of *L. chinense* fruit, may vary depending on the cultivation environment. Thus, this study aims to quantitatively analyze the betaine content in *L. chinense* fruits and identify the correlations between environmental variables and growth characteristics. By doing so, it seeks to support the stable production of medicinal goji berries, which have significant potential in the pharmaceutical industry due to their high value, and to provide foundational data for consistent quality control.

## 2. Results

### 2.1. Soil Physicochemical Properties and Meteorological Variables

The 12 soil physicochemical properties were analyzed (Table S1). As a result of soil analysis, soil texture was identified as sandy clay loam, sandy loam, and loam. For soil pH, the highest value at site 12 is pH 6.99 ± 0.04, and the lowest at site 13 is pH 4.48 ± 0.08. Thirteen sites belong to the acidity range (pH 5.5–6.5) of soil suitable for tree growth, and excluding four sites, the electrical conductivity (EC) value was found to be appropriate with less than 1.0 dS/m [23]. Site 6 had significantly higher levels of organic matter (OM) and total nitrogen (TN) compared to the other sites. The large standard error of available phosphate (AP) prevented making meaningful comparisons between the sites. For cation exchange capacity (CEC), site 7 recorded the highest value at 28.15 ± 1.04 cmol^+^/kg, whereas site 14 had the lowest value at 7.47 ± 0.26 cmol^+^/kg. Twenty sites fell within the appropriate range for tree growth (12–20 cmol^+^/kg). Base saturation (BS) values were highest at 212.96 ± 3.12% at site 5 and lowest at 37.47 ± 1.65% at site 24.

Meteorological data were collected for seven variables (Table S2). Altitude (ALT) ranged from 12 m to 402 m. The annual average temperature (AAT) ranged from 11.1 to 14.5 °C. The annual average maximum temperature (AAMT) ranged from 17.4 to 20.6 °C, and the annual average minimum temperature (AAmT) ranged from 5.5 to 10.0 °C. The highest recorded annual maximum temperature (AMT) was 37.5 °C at site 2, while the lowest annual minimum temperature (AmT) was −23.8 °C at sites 18–22. The total precipitation (TP) reached its peak at sites 12–14 with 1549.1 mm and was at its lowest at site 11 with 942.0 mm.

### 2.2. Growth Characteristics of L. chinense Fruit

Table 1 presents the data on the growth characteristics of *L. chinense* fruit. The length of fruit (LF) was the longest at 26.49 ± 0.53 mm in the Cheongyang 2 area (site 19), and the width of fruit (WF) was the widest at 11.38 ± 0.11 mm in the Sangju area (site 5). The shortest LF was 15.62 ± 0.22 mm in the Buyeo area (15 sites), and the narrowest WF was 7.09 ± 0.88 mm in the Cheongyang 4 areas (21 sites). The aspect ratio (AR) ranged from 0.33 ± 0.00 to 0.53 ± 0.03. As for the fresh weight of fruit (FWF, one fruit), the heaviest in Yeongju area 1 (site 8) at 1.62 ± 0.06 g, and the lightest in site 21 at 0.73 ± 0.05 g. The heavier the FWF, the higher the LF and WF tended to be, and vice versa. The sugar content (SG) of the fruit was the highest in the site 15 at 19.62 ± 1.09 Brix° and the lowest in the Yeongcheon area (site 10) at 11.10 ± 1.02 Brix°.

### 2.3. Validation and Quantification of Betaine

**Figure 1:**
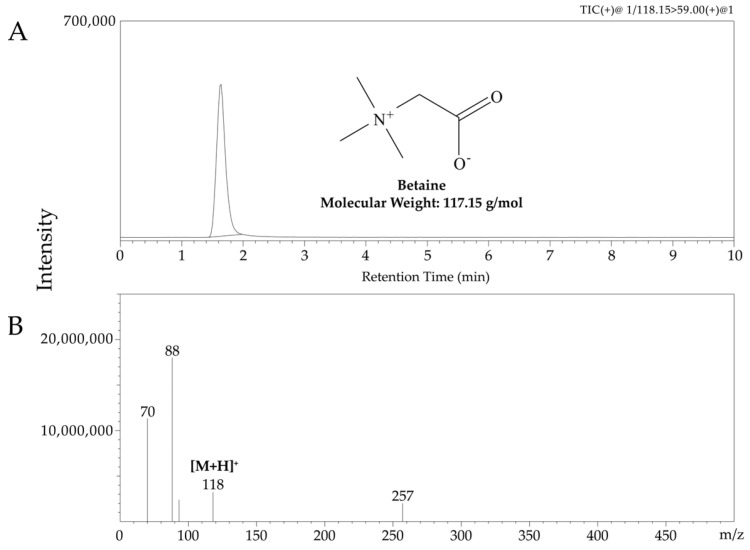
UHPLC-MS/MS data spectrum for betaine. (**A**) Extracted total ion chromatogram at *m*/*z* 118; (**B**) MS/MS spectrum of protonated molecule at *m*/*z* 118. The *m*/*z* 88 peak is the broken bond of the (CH_3_)_2_-N group, and the *m*/*z* 70 peak is thought to be due to the loss of the CH_3_ and OOH groups.

The developed UHPLC-MS/MS methods were validated for quantitative analysis, and the results are presented in Table 2 and Figure 1. The linearity of betaine is shown to be good, with a correlation coefficient exceeding 0.9991. Betaine had a limit of detection (LOD) of 0.01 μg/mL and a quantification (LOQ) value of 0.02 μg/mL. The precision, both intra- and inter-day, was assessed by measuring betaine concentrations at three distinct levels, three times each. The relative standard deviation (RSD) was calculated as the standard deviation divided by the mean, expressed as a percentage. The RSD values ranged from 0.10% to 0.51% for intra-day precision and from 0.08% to 0.22% for inter-day precision, all of which fall within the acceptable limits. These findings confirm that the developed UHPLC-MS/MS analytical method is reliable for quantifying betaine content in *L. chinense* fruits.

**Figure 2:**
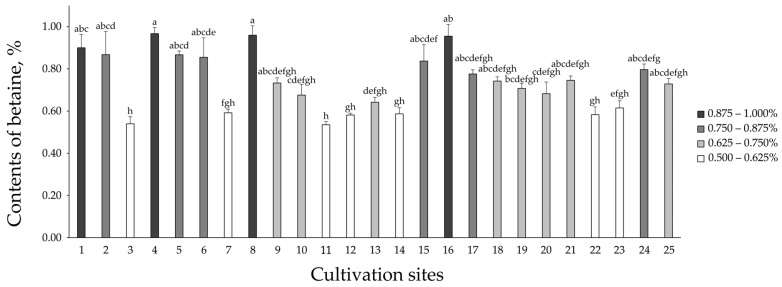
Contents of betaine in the fruit of *Lycium chinense* by 25 different cultivation sites. Error bars represent standard errors. The letters at the top of the bars indicate the Tukey post-hoc test results, indicating a significant difference between the groups (*p* < 0.05).

The 75 fruit samples of *L. chinense* were analyzed using the UHPLC-MS/MS method. Betaine was identified by comparison the peak and retention time of the standard solution with the total ion chromatogram. Betaine was detected at a retention time of 1.625 min. The results of the quantitative analysis are presented in Table S3 and Figure 2. The contents of betaine in samples from 0.540 ± 0.034 to 0.967 ± 0.029%. The betaine content was found to be the highest in the *L. chinense* fruit at site 4.

### 2.4. Correlations among Betaine, Environments, and Fruit Growth

**Figure 3:**
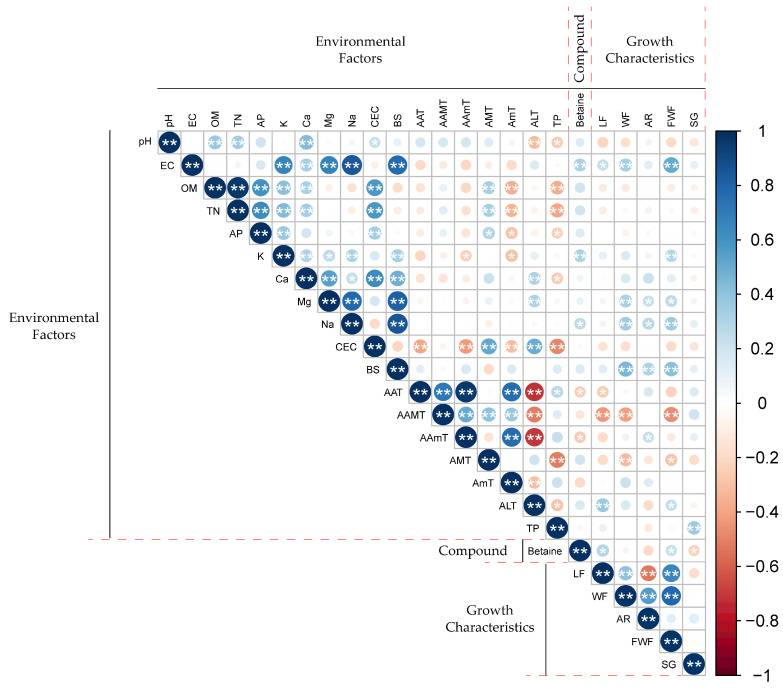
Correlation analysis of betaine, growth characteristics, and environmental variables. * *p* < 0.05, ** *p* < 0.01.

Pearson correlation analysis between contents of betaine in *L. chinense* fruits and environmental data (soil physicochemical properties and meteorological variables) was performed (Tables S4 and S5 and Figure 3). Betaine positively correlated with EC (0.327, *p* = 0.004), K^+^ (0.314, *p* = 0.006), and Na^2+^ (0.259, *p* = 0.025), whereas AAmT (−0.256, *p* = 0.027) and AAT (−0.242, *p* = 0.036) were negatively correlated with contents of betaine. Overall, betaine contents were positively correlated with soil variables and negatively correlated with meteorological variables.

**Figure 4:**
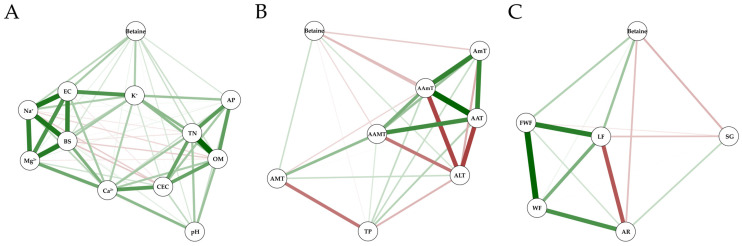
Correlation network model depicting the relationships among betaine, environmental variables ((**A**) soil physicochemical properties, (**B**) meteorological variables), and growth characteristics (**C**). Green lines denote positive correlations, red lines denote negative correlations. The thickness of the edges represents the strength of the relationships.

The correlation among environmental variables, betaine, and growth characteristics of *L. chinense* fruits was visualized through a network model (Figure 4). The node represents each variable and the edge represents the unique association between the variables. The network model (Figure 4A) showed that betaine is positively correlated with soil parameters such as Na^2+^, EC, K^+^, and AP. Also, Figure 4B showed that betaine is negatively correlated with meteorological variables such as AAT, AAmT, and AmT. Correlation analysis between betaine content and fruit growth characteristics of *L. chinense* was also performed (Table S6 and Figure 3). The contents of betaine have a positive correlation with LF (0.294, *p* = 0.010) and FWF (0.238, *p* = 0.040) and a negative correlation with SG (−0.232, *p* = 0.046). The network model (Figure 4C) showed a positive correlation between betaine and FWF and LF, and a negative correlation with SG.

## 3. Discussion

*L. chinense* is a valuable species known for its strong abiotic stress resistance and high nutritional content, making it a popular functional food in oriental medicine [9]. Major active compounds such as polysaccharides, flavonoids, vitamins, and betaine significantly influence the quality and market value of *L. chinense* fruit [24,25]. This study aims to investigate how environmental variables at the cultivation site impact the growth of *L. chinense* fruits and their betaine content.

Soil physicochemical properties (Table S1) of *L. chinense* cultivation sites were compared with plant growth suitability [23]. Thirteen cultivation sites had soil acidity values in the appropriate range (pH 5.5−6.5). EC (<1.0) was found to be suitable in 21 cultivation sites. OM (≥3.0) and TN (≥0.25) were at good levels at 13 and 6 sites, respectively. AP (≥100) was mostly suitable except for two sites. In the case of exchangeable cations, K^+^ was ideal in all cultivation sites and Mg^2+^ in 21 cultivation sites, but most Ca^2+^ and Na^+^ were not within the appropriate range. BS was in the range 37.47–212.96%. Generally, acidification of soil increases the concentration of Al^3+^, which is known to be a toxic substance that interferes with the absorption and movement of nutrients, thereby hindering root development and plant growth [26]. Moreover, high levels of soil OM enhance plant nitrogen uptake by promoting nitrogen mineralization, which in turn boosts crop productivity [27,28]. Additionally, meteorological data were gathered (Table S2). The annual average temperature (AAT) varied between 11.1 °C and 14.5 °C. The highest annual average maximum temperature (AAMT) recorded was 20.6 °C, while the lowest annual average minimum temperature (AAmT) was 5.5 °C. Total precipitation (TP) ranged from 942.0 mm to 1549.1 mm. *L. chinense* thrives in fertile sandy loam with good sunlight, deep soil, and good moisturizing and drainage [29], but is sometimes grown in environments that are not. Environmental variables like altitude, temperature, precipitation, and soil pH interact in complex ways, influencing plant growth and survival [30]. Although studies have been conducted on how plant growth changes depending on the environment, more studies are still needed on its impact on metabolites. Plant metabolites are produced to defend themselves and are induced through various biosynthetic pathways by abiotic and biotic factors [31].

The growth characteristics of *L. chinense* fruits significantly differed depending on the cultivation sites. The largest fruits were observed in the Yeongju1 area (Site 8), while the smallest fruit were recorded in the Cheongyang4 area (Site 21). Analysis of the correlations among growth characteristics showed no significant relationship between fruit size (length, width, and fresh weight) and sugar content (SG) (Figure 3). The quality of fruit, including its size and active compounds, is influenced by various factors such as the soil microbiome, environmental conditions, and genetic factors [32,33,34]. Determining which of these factors has the most significant impact on plant growth is challenging due to the complex and simultaneous interactions of these variables during plant development.

The analytical methods used for the quantification of betaine in *L. chinense* fruits were validated in this study. The validation outcomes, which included linearity, LOD, LOQ, and precision, demonstrated that the analytical method yielded consistent and dependable results. Betaine was detected within 1.625 min using this validated method. Although there were variations among the 25 cultivation sites, the betaine content ranged from 0.535% to 0.967%, beyond the Korean Pharmacopoeia benchmark of 0.5%. These results are consistent with previously reported betaine content in Korean gugija, which ranged from 6.49 to 10.77 mg/g. The analysis indicated that betaine content in *L. chinense* fruits is positively correlated with length of fruit (LF) and fresh weight of fruit (FWF), while it has a negative correlation with sugar content (SG). Plant growth depends on various factors such as sugars, protein synthesis, ATP, reducing power, and a reducing cellular environment. Betaine influences these factors directly or indirectly by affecting its own synthesis, transport, and accumulation, and it positively affects plant growth and development under stress conditions [35].

The concentration of betaine in *L. chinense* exhibits significant fluctuations as a result of varying environmental conditions, highlighting the adaptability and resilience of this fruit. Betaine, a compound found in both animals and plants, plays several vital roles. It serves as a critical osmoprotectant that aids the survival and growth of plants during abiotic stress conditions [22]. In environments with high salinity, plants experience increased physiological stress. Many species, such as *L. chinense*, respond to such salt stress by ramping up the production of betaine. Our research results similarly demonstrated that increased betaine content was positively correlated with higher soil electrical conductivity, exchangeable potassium, and sodium levels. We believe this is because exchangeable potassium and sodium directly influence electrical conductivity, and higher electrical conductivity values are indicative of higher soil salinity. Betaine acts as an osmoprotectant by regulating intracellular osmotic pressure, maintaining water balance within cells, and ensuring membrane stability. These functions are crucial as they prevent cellular damage and maintain cellular integrity and function under stressful conditions. Research by Sakamoto and Murata (2002) underscores that betaine significantly enhances plant survival under stress by adjusting intracellular osmotic conditions to more favorable levels, thereby mitigating the harmful effects of salts on cellular activities [36]. Our results also indicated that betaine content increased when the annual average and annual average minimum temperatures were lower. Cold environments present considerable challenges to plant health. The accumulation of betaine in response to low temperatures is a key defense strategy. It works by preventing the formation of ice crystals within cells and maintaining the fluidity of cellular membranes. These actions help mitigate the impacts of freezing and enable the plant cells to continue functioning despite the cold stress. In a study by Annunziata et al. (2017), it was observed that in response to cold stress, plants like durum wheat significantly increase their internal levels of betaine and other osmoprotectants, illustrating how crucial these compounds are for cold resistance [37].

Furthermore, the application of these findings to the cultivation and management of *L. chinense* indicates the necessity to consider and adapt to environmental variables actively. Understanding how betaine levels change in response to these factors enables farmers and agriculturalists to develop more effective strategies for crop management and improvement. For example, adjustments in watering schedules, soil salinity levels, and exposure to cold can be fine-tuned to create optimal conditions for plants to produce ideal levels of betaine, thus enhancing their nutritional and medicinal values [38,39]. Additionally, in-depth research into the genetic pathways and environmental interactions that regulate betaine synthesis in *L. chinense* could lead to significant breakthroughs in agricultural practices [40]. Potential developments include creating cultivars that are more stress-resistant or that consistently produce high levels of betaine, regardless of environmental variations.

Therefore, the importance of betaine in managing plant stress responses and its direct impact on the healthfulness of *L. chinense* underscores the broader significance of plant physiology research and agricultural biotechnology. The field is open to continuous exploration and promises to provide significant benefits not only for cultivating *L. chinense*, but also for other crops that can benefit from improved stress resistance and enhanced physiological functions.

## 4. Materials and Methods

### 4.1. Sampling of Plant Material and Chemicals

**Figure 5:**
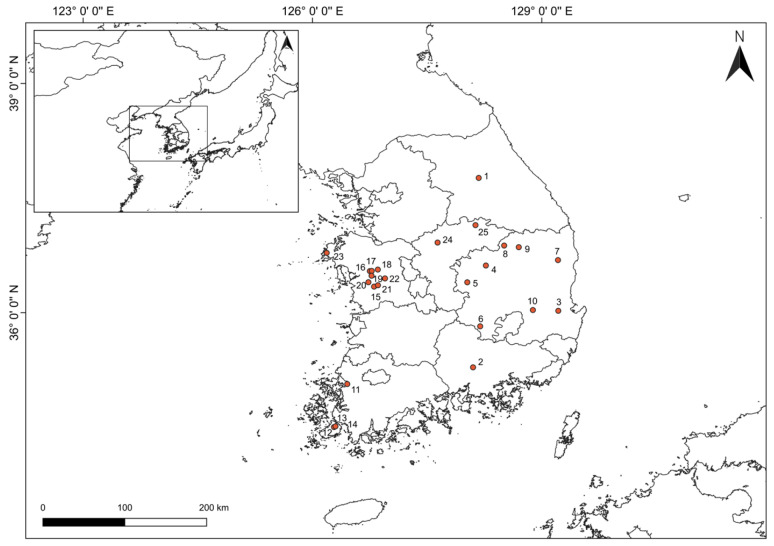
Geographical information for the cultivated fruit samples of *Lycium chinense* from the various provinces. Each number is a number assigned to cultivation sites.

In November 2021, from 25 cultivation sites spanning 17 regions in South Korea, we collected 75 samples of *L. chinense* fruits (specimen numbers: FMRC-B2111001~B2111075) and soil samples from these locations (Table S7 and Figure 5). The collected voucher specimens have been deposited in the herbarium of the Forest Medicinal Resources Research Center (FMRC), which is affiliated with the National Institute of Forest Science in Korea. The fruits moisture content of *L. chinense* averaged 84.54 ± 0.69%. Five growth characteristics were measured and calculated, including the length and width of the fruit (LF and WF), aspect ratio (AR), fresh weight of fruit (FWF), and sugar content (SG). For these measurements, digital calipers manufactured by Mitutoyo Co., based in Kawasaki, Japan (model 500-182-30), were used to determine LF and WF. The FWF was measured using an electronic scale produced by HANSUNG Instrument Co. from Gwangmyeong, Republic of Korea (model HS3200S). The SG was assessed using a refractometer provided by ATAGO Co., Ltd., located in Tokyo, Japan (model PR-101α). HPLC-grade acetonitrile, ethanol, and water, obtained from J.T. Baker, a subsidiary of Avantor, Inc., headquartered in Radnor, PA, USA, were used in the analysis without further purification.

### 4.2. Sample and Standard Preparation

The collected samples were rinsed with water and then lyophilized. After lyophilization, the samples were ground using a grinder (model KSP-35) from Korea Medi Co., Ltd., Daegu, Republic of Korea, and stored at −18 °C until analysis.

For sample preparation, one gram of the dried powder was extracted in 100 mL of 80% ethanol using ultrasonication in a 200 mL flask at room temperature for one hour. The ultrasonic bath (model JAC-5020) used was manufactured by KODO in Hwaseong, Korea, with a power output of 350 W and a frequency of 40 kHz. After extraction, the mixture was centrifuged at 1763× *g* for 15 min using a Labogene UM-1248 centrifuge from Bio Medical Science Co., Ltd., Seoul, Republic of Korea. The supernatant was then filtered through ADVENTEC^®^ No. 2 filter paper from Toyo Roshi Kaisha, Ltd., Tokyo, Japan. The samples were further filtered through 0.2-μm PTFE syringe filters (model 6784-1302) from Whatman Co., Maidstone, UK. For betaine analysis, standard stock solutions were prepared by diluting the stock with ethanol to achieve concentrations ranging from 0.3125 to 5 µg/mL.

### 4.3. UHPLC-MS/MS Analysis

Betaine analysis was performed using a UHPLC-MS/MS system. The chromatographic separation was conducted with a Shimadzu LC-30AD UHPLC system, which includes two LC-30AD pumps, a SIL-30AC auto-sampler, a DGU-20A5R degasser, a CTO-20A column oven, and an SPD-M20A UV–vis detector, all manufactured by Shimadzu, Kyoto, Japan. The system was paired with a Shimadzu LCMS-8050 triple quadrupole mass spectrometer, also from Shimadzu, featuring an electrospray ionization (ESI) source. For the liquid chromatography, an Acquity UPLC BEH HILIC column (2.1 mm × 100 mm, 1.7 μm, 130 Å) from Waters Co., Milford, MA, USA, was used with 98% acetonitrile as the mobile phase under isocratic conditions. The column was maintained at 30 °C, with an injection volume of 1 μL and a flow rate of 0.3 mL/min. The ESI source was operated in positive ion mode, with nebulizing and drying gas flows set to 3 L/min and 15 L/min, respectively. The desolvation line (DL) and heat block temperatures were set at 240 °C and 400 °C, respectively. Mass spectrometry data were acquired in multiple reaction monitoring (MRM) modes with positive ionization, where the precursor ion of betaine at *m*/*z* 118.15 was transitioned to the product ion at *m*/*z* 59.00 with a collision energy of −15 eV and a dwell time of 100 ms. Chromatographic analyses were controlled and data were acquired using LabSolutions software (version 5.82) from Shimadzu. Each sample was analyzed in triplicate, and the results were reported as mean values.

The analytical method was validated by the International Conference on Harmonization (ICH) guideline Q2 [41], focusing on linearity, LOD, LOQ, and precision (both intra-day and inter-day). Betaine calibration curves were generated using standard solutions across five different concentrations within the range of 0.3125–5 μg/mL. The LOD and LOQ were established based on signal-to-noise ratios of 3.3 and 10, respectively. Precision was assessed by calculating the relative standard deviations (RSD, %) for repeatability, as well as intra-day (within the same day) and inter-day (across three consecutive days) variations. Samples were analyzed in triplicate at three different concentrations, and the results were expressed using the relative standard deviations to the mean values.

### 4.4. Soil Analysis and Collecting Meteorological Data

At the cultivation sites, soil samples were taken from a depth of 20 cm, following the removal of the surface layer. The samples were left to air-dry at room temperature and then sifted through a 2 mm sieve before storage. Soil was classified into 12 classes of soil texture classification according to the soil survey manual of the United States Department of Agriculture (USDA). The analysis of soil physicochemical properties, including soil acidity, electrical conductivity (EC), organic matter (OM), total nitrogen (TN), available phosphate (AP), exchangeable cations (K^+^, Ca^2+^, Mg^2+^, and Na^+^), and cation exchange capacity (CEC), was performed following the protocol specified in the analysis manual issued by the Rural Development Administration (RDA) of Korea [42]. Base saturation (BS) was determined as the percentage of the sum of exchangeable cations divided by the CEC.

Data on weather conditions at the cultivation site, such as annual average temperature (AAT), annual average maximum temperature (AAMT), annual average minimum temperature (AAmT), annual maximum temperature (AMT), annual minimum temperature (AmT), and total precipitation (TP), were obtained from the Korea Meteorological Administration’s open data portal (data.kma.go.kr, accessed on 20 September 2022).

### 4.5. Statistical Analysis

Statistical analyses were performed using SPSS software, specifically Version 26 of IBM SPSS Statistics. The results were presented as mean ± standard error (S.E.), and the analyses were conducted at a 5% significance level. Differences between groups were evaluated using multivariate analysis of variance (MANOVA) and Tukey’s multiple comparison test. Pearson’s correlation coefficient was employed to examine the relationships among growth characteristics, betaine content in *L. chinense* fruits, and environmental variables at the cultivation site. Correlation and network models were visualized using the ggcorrplot2 and qgraph packages in R Studio (Version R 4.2.3) from Posit Inc., Boston, MA, USA.

## 5. Conclusions

In this study, we focused on how betaine of *L. chinense* fruit was affected by the growth environment and correlated with fruit growth. *L. chinense* fruit was collected and analyzed throughout Korea. Consequently, betaine demonstrated a positive correlation with EC, K^+^, and Na^+^, while showing a negative correlation with AAT and AAmT. Furthermore, betaine was found to positively correlate with LF and FWF, and negatively correlate with SG. It emphasizes the important role of betaine in improving plant resilience to abiotic stresses such as high salt and low temperature. As a result, agricultural experts can optimize cultivation methods to produce goji berries with higher nutritional and medicinal values by understanding the relationship between environmental variables and betaine synthesis.

## Figures and Tables

**Table 1:** Growth characteristics of *Lycium chinense* fruit by 25 different cultivation sites.

Cultivation Sites (*n* = 3)	LF	WF	AR	FWF	SG
	(mm)	(mm)	(α:1)	(g)	(Brix°)
1	26.09 ± 0.28 ^ab^	10.12 ± 0.43 ^ab^	0.39 ± 0.01 ^def^	1.44 ± 0.15 ^abcd^	14.02 ± 0.44 ^defg^
2	19.37 ± 0.65 ^fgh^	7.68 ± 0.19 ^defg^	0.40 ± 0.02 ^bcdef^	0.81 ± 0.08 ^ghi^	16.14 ± 0.40 ^abcde^
3	17.29 ± 0.34 ^hi^	7.55 ± 0.30 ^defg^	0.44 ± 0.02 ^abcdef^	0.82 ± 0.06 ^fghi^	18.86 ± 0.50 ^abc^
4	23.22 ± 0.30 ^bcde^	8.99 ± 0.40 ^bcdefg^	0.39 ± 0.02 ^def^	1.24 ± 0.07 ^abcdef^	15.20 ± 1.41 ^bcdefg^
5	22.09 ± 0.59 ^def^	11.38 ± 0.11 ^a^	0.52 ± 0.01 ^abc^	1.55 ± 0.05 ^ab^	16.29 ± 0.76 ^abcde^
6	21.03 ± 0.29 ^efg^	8.66 ± 0.05 ^bcdefg^	0.41 ± 0.01 ^abcdef^	0.95 ± 0.00 ^efghi^	15.69 ± 0.94 ^abcdef^
7	19.41 ± 0.77 ^fgh^	8.67 ± 0.51 ^bcdefg^	0.45 ± 0.01 ^abcdef^	0.89 ± 0.11 ^fghi^	15.23 ± 0.82 ^bcdefg^
8	24.98 ± 1.01 ^abcd^	10.29 ± 0.05 ^ab^	0.42 ± 0.02 ^abcdef^	1.62 ± 0.06 ^a^	14.13 ± 0.82 ^defg^
9	25.32 ± 0.84 ^abc^	8.47 ± 0.16 ^bcdefg^	0.34 ± 0.02 ^f^	1.12 ± 0.03 ^cdefghi^	13.80 ± 0.76 ^defg^
10	22.13 ± 0.13 ^def^	8.80 ± 0.30 ^bcdefg^	0.40 ± 0.01 ^bcdef^	0.96 ± 0.11 ^efghi^	11.10 ± 1.02 ^g^
11	19.16 ± 0.16 ^fgh^	10.00 ± 0.44 ^ab^	0.52 ± 0.03 ^ab^	1.37 ± 0.06 ^abcde^	13.69 ± 0.48 ^defg^
12	22.05 ± 0.47 ^def^	9.52 ± 0.41 ^abcde^	0.43 ± 0.01 ^abcdef^	1.09 ± 0.11 ^defghi^	17.41 ± 1.24 ^abcd^
13	22.12 ± 0.57 ^def^	8.87 ± 0.16 ^bcdefg^	0.40 ± 0.00 ^abcdef^	0.97 ± 0.06 ^efghi^	16.03 ± 0.62 ^abcde^
14	22.44 ± 0.91 ^cde^	10.08 ± 0.32 ^ab^	0.45 ± 0.03 ^abcdef^	1.18 ± 0.05 ^bcdefg^	18.80 ± 0.75 ^abc^
15	15.62 ± 0.22 ^i^	7.88 ± 0.13 ^cdefg^	0.51 ± 0.01 ^abcde^	0.79 ± 0.03 ^hi^	19.62 ± 1.09 ^a^
16	22.71 ± 0.39 ^cde^	7.46 ± 0.16 ^fg^	0.33 ± 0.01 ^f^	1.01 ± 0.03 ^efghi^	12.89 ± 1.12 ^efg^
17	22.03 ± 0.32 ^def^	9.56 ± 0.45 ^abcd^	0.43 ± 0.01 ^abcdef^	1.21 ± 0.11 ^abcdefg^	12.58 ± 0.28 ^efg^
18	18.15 ± 0.32 ^ghi^	9.52 ± 0.41 ^abcde^	0.53 ± 0.03 ^a^	1.20 ± 0.04 ^bcdefgh^	16.18 ± 0.67 ^abcde^
19	26.49 ± 0.51 ^a^	8.74 ± 0.24 ^bcdefg^	0.33 ± 0.00 ^f^	1.51 ± 0.10 ^abc^	17.20 ± 0.13 ^abcd^
20	19.31 ± 0.10 ^fgh^	7.39 ± 0.39 ^fg^	0.38 ± 0.02 ^ef^	0.75 ± 0.04 ^i^	18.91 ± 0.44 ^ab^
21	16.89 ± 0.50 ^hi^	7.09 ± 0.88 ^g^	0.42 ± 0.06 ^abcdef^	0.73 ± 0.05 ^i^	11.71 ± 0.87 ^fg^
22	19.19 ± 0.53 ^fgh^	7.48 ± 0.29 ^efg^	0.39 ± 0.03 ^cdef^	0.87 ± 0.04 ^fghi^	14.96 ± 0.05 ^bcdefg^
23	19.38 ± 0.15 ^fgh^	9.88 ± 0.59 ^abc^	0.51 ± 0.03 ^abcd^	0.96 ± 0.07 ^efghi^	14.74 ± 0.40 ^cdefg^
24	22.79 ± 1.12 ^cde^	9.36 ± 0.54 ^abcdef^	0.42 ± 0.04 ^abcdef^	1.13 ± 0.06 ^bcdefghi^	15.77 ± 0.54 ^abcdef^
25	23.87 ± 0.11 ^abcde^	11.07 ± 0.23 ^a^	0.46 ± 0.01 ^abcde^	1.46 ± 0.12 ^abcd^	16.12 ± 0.45 ^abcde^

LF, length of fruit; WF, width of fruit; AR, aspect ratio; FWF, fresh weight of fruit; SG, sugar contents. Mean ± Standard Error (S.E.) values are presented. Mean values labeled with distinct letters denote significant differences as determined by Tukey’s test (*p* < 0.05).

**Table 2:** Linear regression, LOD, LOQ of betaine.

Parameters	Value
Linearity range (µg/mL)	0.3125–5
Regression equation	Y = 1,053,004.8258 X + 88,006.7500
Regression coefficient (*r*^2^)	0.9991
LOD, limit of detection (µg/mL)	0.01
LOQ, limit of quantification (µg/mL)	0.02
Precision, Intra-day ^a^ (%RSD)	0.10–0.51
Precision, Inter-day ^b^ (%RSD)	0.08–0.22

^a^ Sample analyzed three times within a single day (*n* = 3); ^b^ Sample analyzed three times over three consecutive days (*n* = 3); RSD stands for relative standard deviation.

## Data Availability

The data presented in this study are available under permission from the corresponding author on reasonable request.

## Supplementary Materials

The following supporting information can be downloaded at: https://www.mdpi.com/article/10.3390/plants13162316/s1, Table S1: Soil physicochemical properties of 25 different *Lycium chinense* cultivation sites; Table S2: Meteorological data of 25 different *Lycium chinense* cultivation sites; Table S3: The contents of betaine in *Lycium chinense* fruit from 25 different cultivation sites; Table S4: Pearson’s correlation coefficient between contents of betaine and soil physicochemical properties of *Lycium chinense* cultivation sites; Table S5: Pearson’s correlation coefficient between contents of betaine and meteorological properties of *Lycium chinense* cultivation sites; Table S6: Pearson’s correlation coefficient between contents of betaine and growth characteristics of *Lycium chinense* fruit; Table S7: Geographic information about the cultivation sites where fruits of *Lycium chinense* were collected in South Korea.
