# Supplementary material for: Study on Betaine and Growth Characteristics of Lycium chinense Mill. in Different Cultivation Environments in South Korea
Source: Plants (Basel). 2024 Aug 20;13(16):2316. doi: 10.3390/plants13162316 (PMC11359574; doi:10.3390/plants13162316)
Supplement: Supplementary file 1 [file plants-13-02316-s001.zip › plants-3104477-supplementary.pdf]

SUPPLEMENTARY DATA

Study on Betaine and Growth Characteristics of *Lycium chinense* Mill. in Different Cultivation Environments in South Korea

Hyejung Cho [1,2,†], Dong Hwan Lee [1,†], Dae Hui Jeong [1], Jun Hyuk Jang [1], Yonghwan Son [1], Sun-Young Lee [1] and Hyun-Jun Kim [1,*]

[1] Forest Medicinal Resources Research Center, National Institute of Forest Science, Yeongju-si 36040, Republic of Korea; whgpwjd@korea.kr (H.C.); leedh0419@korea.kr (D.H.L.); najdhda@korea.kr (D.H.J.); wnseldu123@korea.kr (J.H.J.); thsdydghks@korea.kr (Y.S.); nararawood@korea.kr (S.-Y.L.)
[2] School of Environmental Engineering, University of Seoul, Seoul 02504, Republic of Korea
* Correspondence: mind4938@korea.kr
† These authors contributed equally to this work.

Table S1. Soil physicochemical properties of 25 different *Lycium chinense* cultivation sites.

Cultivation Sites (n=3)	Soil texture	pH [1:5]	EC (dS/m)	OM (%)	TN (%)	AP (mg/kg)	Exchangeable cation K^+ (cmol⁺/kg)	Ca^{2+}	Mg^{2+}	Na^+	CEC (cmol⁺/kg)	BS (%)
1	SCL	5.63±0.01^{cdefgh}	0.45±0.02^{d}	3.95±0.21^{bcd}	0.25±0.03^{bcd}	1424.81±1210.24^{a}	0.91±0.12^{bc}	8.42±0.14^{bcdefgh}	2.41±0.07^{cdef}	0.18±0.0^{b}	16.65±0.19^{cde}	71.61±1.13^{bcd}
2	SCL	5.85±0.01^{abcdefg}	0.13±0.01^{d}	2.98±0.21^{bcdefg}	0.18±0.00^{bcdef}	1238.14±630.38^{a}	0.78±0.15^{bc}	5.05±0.20^{fgh}	0.90±0.02^{f}	0.02±0.00^{b}	14.86±0.38^{def}	45.37±1.24^{cd}
3	L	6.32±0.18^{abcde}	0.14±0.02^{d}	3.09±0.18^{bcdefg}	0.20±0.01^{bcde}	1072.53±509.12^{a}	0.57±0.25^{bc}	6.69±0.88^{cdefgh}	1.73±0.24^{def}	0.05±0.02^{b}	15.04±0.12^{def}	60.20±8.44^{bcd}
4	SCL	6.74±0.09^{abc}	0.13±0.01^{d}	2.79±0.39^{bcdefg}	0.18±0.01^{bcdef}	1159.43±344.89^{a}	0.60±0.06^{bc}	11.77±0.71^{abc}	2.22±0.06^{cdef}	0.03±0.00^{b}	16.68±0.23^{cdef}	87.72±4.71^{bcd}
5	SCL	6.03±0.33^{abcdef}	2.80±0.61^{a}	1.03±0.31^{ghi}	0.14±0.01^{def}	844.25±633.19^{a}	0.99±0.52^{bc}	10.90±0.34^{bcde}	8.68±0.93^{a}	3.85±0.64^{a}	11.44±0.94^{fg}	212.96±3.12^{a}
6	SL	6.20±0.18^{abcdef}	0.62±0.29^{d}	6.93±1.34^{a}	0.43±0.08^{a}	1827.17±866.95^{a}	1.91±0.80^{ab}	10.44±0.48^{bcdef}	4.14±0.69^{bc}	0.25±0.17^{b}	23.15±1.98^{b}	73.02±9.96^{bcd}
7	SL	5.13±0.08^{fghi}	0.09±0.01^{d}	1.24±0.07^{fghi}	0.13±0.00^{def}	327.27±8.58^{a}	0.39±0.02^{c}	16.79±2.24^{a}	5.58±0.58^{b}	0.07±0.01^{b}	28.15±1.04^{a}	80.61±7.09^{bcd}
8	SCL	5.20±0.31^{efghi}	1.90±0.26^{abc}	2.79±0.11^{bcdefg}	0.18±0.00^{bcdef}	647.41±80.03^{a}	2.62±0.35^{a}	9.74±1.75^{bcdefg}	3.31±0.59^{bcde}	0.90±0.17^{b}	15.40±0.25^{cdef}	108.19±19.22^{d}
9	SCL	6.79±0.09^{ab}	0.44±0.22^{d}	3.11±0.41^{bcdefg}	0.20±0.01^{bcde}	793.74±96.12^{a}	0.76±0.23^{bc}	8.92±0.19^{bcdefgh}	2.16±0.57^{cdef}	0.11±0.06^{b}	16.53±0.18^{cde}	72.27±6.55^{bcd}
10	SCL	5.84±0.59^{bcdefg}	0.08±0.01^{d}	1.56±0.12^{efghi}	0.13±0.01^{def}	404.65±64.22^{a}	0.34±0.02^{c}	6.84±1.84^{cdefgh}	2.19±0.50^{cdef}	0.06±0.01^{b}	13.79±0.59^{ef}	67.97±15.60^{bcd}
11	SCL	6.09±0.29^{abcdef}	0.10±0.01^{d}	1.82±0.32^{defghi}	0.15±0.01^{cdef}	667.10±35.99^{a}	0.66±0.06^{bc}	6.52±0.66^{cdefgh}	1.65±0.15^{def}	0.16±0.02^{b}	14.15±0.78^{ef}	63.55±5.46^{bcd}
12	SCL	6.99±0.04^{a}	0.12±0.01^{d}	2.52±0.31^{bcdefgh}	0.17±0.01^{bcdef}	639.84±45.41^{a}	0.61±0.06^{bc}	10.60±0.77^{bcdef}	1.45±0.07^{def}	0.10±0.01^{b}	15.37±0.52^{cdef}	82.96±2.67^{bcd}
13	L	4.48±0.08^{i}	0.14±0.01^{d}	0.25±0.06^{hi}	0.08±0.02^{ef}	94.03±20.07^{a}	0.77±0.11^{bc}	3.99±0.04^{h}	2.45±0.27^{cdef}	0.07±0.02^{b}	7.56±0.64^{g}	97.73±9.35^{bc}
14	L	4.61±0.10^{hi}	0.08±0.02^{d}	0.19±0.01^{i}	0.07±0.00^{f}	77.44±2.74^{a}	0.33±0.02^{c}	3.71±0.37^{h}	2.45±0.13^{cdef}	0.11±0.01^{b}	7.47±0.26^{g}	88.14±3.23^{bcd}
15	L	6.81±0.15^{ab}	1.24±0.72^{bcd}	4.53±0.06^{b}	0.28±0.01^{b}	983.60±106.41^{a}	1.92±0.56^{ab}	11.66±1.61^{abc}	2.42±0.72^{cdef}	0.98±0.59^{b}	16.57±0.95^{cde}	105.55±27.66^{b}
16	SCL	6.36±0.03^{abcd}	0.14±0.02^{d}	2.23±0.16^{cdefghi}	0.18±0.01^{bcdef}	1099.33±89.23^{a}	0.89±0.08^{bc}	5.54±0.18^{efgh}	1.05±0.07^{ef}	0.03±0.00^{b}	15.05±0.50^{def}	50.00±1.47^{cd}
17	SCL	6.39±0.05^{abcd}	0.30±0.01^{d}	7.09±0.56^{a}	0.45±0.01^{a}	2039.59±111.70^{a}	1.30±0.16^{abc}	11.74±0.46^{abc}	2.83±0.19^{cdef}	0.06±0.00^{b}	19.32±0.59^{bc}	82.62±1.95^{bcd}
18	SCL	5.96±0.09^{abcdef}	0.70±0.21^{cd}	3.76±0.36^{bcde}	0.29±0.04^{b}	2053.28±224.61^{a}	1.39±0.23^{abc}	8.62±0.75^{bcdefgh}	2.26±0.21^{cdef}	0.18±0.08^{b}	16.42±1.13^{cde}	75.81±4.19^{bcd}
19	SCL	5.62±0.10^{cdefghi}	2.09±0.33^{ab}	3.30±0.78^{bcdefg}	0.22±0.03^{bcd}	1487.64±350.92^{a}	2.00±0.30^{ab}	11.45±1.40^{abcd}	3.46±0.51^{bcd}	0.66±0.16^{b}	16.61±0.97^{cde}	106.74±16.01^{b}
20	SCL	5.58±0.16^{defghi}	0.24±0.09^{d}	3.39±0.26^{bcdef}	0.21±0.01^{bcd}	945.74±84.63^{a}	0.78±0.20^{bc}	5.88±1.20^{defgh}	2.48±0.69^{cdef}	0.19±0.09^{b}	16.47±0.69^{cde}	56.53±13.00^{bcd}
21	SCL	6.44±0.30^{abcd}	0.42±0.14^{d}	3.33±0.42^{bcdefg}	0.20±0.01^{bcd}	354.63±79.73^{a}	1.06±0.30^{bc}	8.16±1.70^{bcdefgh}	1.77±0.45^{def}	0.15±0.02^{b}	16.31±0.49^{cde}	67.47±13.12^{bcd}
22	SCL	5.07±0.12^{fghi}	0.32±0.02^{d}	3.94±0.51^{bcd}	0.25±0.03^{bcd}	1540.41±50.94^{a}	0.95±0.14^{bc}	6.09±0.51^{cdefgh}	2.53±0.20^{cdef}	0.21±0.03^{b}	17.89±0.52^{cde}	54.67±0.59^{bcd}
23	SCL	6.83±0.30^{ab}	0.17±0.02^{d}	4.39±0.34^{bc}	0.27±0.02^{bc}	1055.06±81.35^{a}	0.85±0.11^{bc}	13.07±1.77^{ab}	2.38±0.40^{cdef}	0.06±0.01^{b}	18.47±0.33^{cd}	88.58±11.35^{bcd}
24	SCL	5.36±0.29^{defghi}	0.12±0.01^{d}	2.43±0.24^{bcdefghi}	0.17±0.01^{bcdef}	479.07±120.05^{a}	0.38±0.08^{c}	4.15±0.25^{gh}	1.11±0.07^{ef}	0.04±0.00^{b}	15.12±0.18^{def}	37.47±1.65^{d}
25	SCL	4.78±0.05^{ghi}	0.66±0.15^{cd}	3.38±0.28^{bcdef}	0.21±0.01^{bcd}	1037.15±93.25^{a}	1.80±0.18^{abc}	7.26±0.31^{cdefgh}	1.60±0.08^{def}	0.07±0.01^{b}	16.31±1.30^{cde}	66.23±2.86^{bcd}

SCL, sandy clay loam; SL, sandy loam; L, loam; EC, electrical conductivity; OM, organic matter; TN, total nitrogen; AP, available phosphate; CEC, cation exchange capacity; BS, base saturation. Mean ± Standard Error (S.E.) values are presented. Mean values labeled with distinct letters denote significant differences as determined by Tukey's test ($p < 0.05$).

Table S2. Meteorological data of 25 different *Lycium chinense* cultivation sites

Cultivation Sites	AAT (°C)	AAMT (°C)	AAmT (°C)	AMT (°C)	AmT (°C)	TP (mm)
1	11.8	18.2	6.5	36.9	-21.5	950.3
2	14.1	20.5	8.5	37.5	-15.4	1395.0
3	13.9	20.0	8.6	36.6	-12.8	1112.1
4	12.5	18.2	7.5	34.7	-16.7	1367.3
5	13.2	19.0	8.2	34.7	-16.3	1230.8
6	13.6	20.6	7.7	36.6	-17.2	1159.0
7	12.0	17.8	6.6	34.4	-17.0	1346.5
8	11.9	18.4	6.2	34.1	-18.9	1199.0
9	13.3	19.4	7.9	36.4	-13.6	1094.2
10	14.1	19.2	9.7	34.2	-14.4	942.0
11	14.5	19.3	10.0	33.3	-13.7	1549.1
12	14.5	19.3	10.0	33.3	-13.7	1549.1
13	13.3	19.5	8.1	34.9	-21.2	1123.4
14	13.5	19.0	8.7	35.7	-18.6	1024.5
15	13.5	19.0	8.7	35.7	-18.6	1024.5
16	12.1	18.9	6.4	35.0	-23.8	1253.0
17	12.1	18.9	6.4	35.0	-23.8	1253.0
18	12.1	18.9	6.4	35.0	-23.8	1253.0
19	12.1	18.9	6.4	35.0	-23.8	1253.0
20	13.4	18.1	9.4	35.0	-16.6	1060.0
21	11.1	17.8	5.5	33.8	-22.5	1209.0
22	11.2	17.4	5.6	34.0	-20.7	1162.2
23	11.8	18.2	6.5	36.9	-21.5	950.3
24	14.1	20.5	8.5	37.5	-15.4	1395.0
25	13.9	20.0	8.6	36.6	-12.8	1112.1

Table S3. The contents of betaine in *Lycium chinense* fruit from 25 different cultivation sites.

Cultivation Sites (n=3)	Betaine (%)
1	0.900 ± 0.063[abc]
2	0.868 ± 0.109[abcd]
3	0.540 ± 0.034[h]
4	0.967 ± 0.029[a]
5	0.867 ± 0.018[abcd]
6	0.855 ± 0.092[abcde]
7	0.592 ± 0.017[fgh]
8	0.960 ± 0.044[a]
9	0.733 ± 0.025[abcdefgh]
10	0.676 ± 0.051[cdefgh]
11	0.535 ± 0.016[h]
12	0.581 ± 0.009[gh]
13	0.642 ± 0.024[defgh]
14	0.587 ± 0.030[gh]
15	0.837 ± 0.078[abcdef]
16	0.955 ± 0.055[ab]
17	0.776 ± 0.020[abcdefgh]
18	0.742 ± 0.020[abcdefgh]
19	0.707 ± 0.025[bcdefgh]
20	0.682 ± 0.055[cdefgh]
21	0.746 ± 0.021[abcdefgh]
22	0.583 ± 0.037[gh]
23	0.615 ± 0.034[efgh]
24	0.797 ± 0.027[abcdefg]
25	0.729 ± 0.026[abcdefgh]

Mean ± Standard Error (S.E.) values are presented. Mean values labeled with distinct letters denote significant differences as determined by Tukey's test ($p < 0.05$).

Table S4. Pearson's correlation coefficient between contents of betaine and soil physicochemical properties of *Lycium chinense* cultivation sites

	Correlation coefficient (r) [a]										
	pH	EC	OM	TN	AP	K^+	Ca^{2+}	Mg^{2+}	Na^+	CEC	BS
Betaine	0.141	0.327**	0.197	0.191	0.149	0.314**	0.077	0.091	0.259*	0.033	0.157
	(0.227)	(0.004)	(0.090)	(0.100)	(0.204)	(0.006)	(0.511)	(0.439)	(0.025)	(0.775)	(0.179)

[a] Correlation coefficient (r) written is significantly correlated between the variables compared. Positive values denote positive correlation and negative values denote negative correlation. Values in bracket means p value (** $p < 0.01$, * $p < 0.05$)

Table S5. Pearson's correlation coefficient between contents of betaine and meteorological properties of *Lycium chinense* cultivation sites

	Correlation coefficient (r) [a]						
	AAT	AAMT	AAmT	AMT	AmT	ALT	TP
Betaine	-0.242*	-0.139	-0.256*	0.197	-0.198	0.169	-0.058
	(0.036)	(0.234)	(0.027)	(0.089)	(0.089)	(0.147)	(0.623)

[a] Correlation coefficient (r) written is significantly correlated between the variables compared. Positive values denote positive correlation and negative values denote negative correlation. Values in bracket means p value (** $p < 0.01$, * $p < 0.05$)

Table S6. Pearson's correlation coefficient between contents of betaine and growth characteristics of *Lycium chinense* fruit

	Correlation coefficient (r) [a]				
	LF	WF	AR	FWF	SG
Betaine	0.294*	0.060	-0.197	0.238*	-0.232*
	(0.010)	(0.607)	(0.090)	(0.040)	(0.046)

[a] Correlation coefficient (r) written is significantly correlated between the variables compared. Positive values denote positive correlation and negative values denote negative correlation. Values in bracket means p value (** $p < 0.01$, * $p < 0.05$).

Table S7. Geographic information about the cultivation sites where fruits of *Lycium chinense* were collected in South Korea

Cultivation sites	name of sites	Altitude (m)	N (latitude)	E (longitude)
1	Hongcheon-gun	275	37.763333	128.174722
2	Jinju-si	27	35.284444	128.100556
3	Gyeongju-si	50	36.024444	129.215278
4	Mungyeong-si	100	36.616944	128.268333
5	Sangju-si	110	36.397500	128.025000
6	Seongju-gun	122	35.821389	128.193889
7	Yeongyang-gun	402	36.688056	129.211389
8	Yeongju-si 1	231	36.878333	128.507500
9	Yeongju-si 2	218	36.856944	128.698611
10	Yeongcheon-si	97	36.034167	128.883333
11	Muan-gun	22	35.065556	126.455000
12	Jindo-gun 1	12	34.502778	126.281389
13	Jindo-gun 2	32	34.511944	126.301389
14	Jindo-gun 3	32	34.511944	126.301389
15	Buyeo-gun	84	36.340833	126.805833
16	Yesan-gun 1	55	36.543889	126.750556
17	Yesan-gun 2	30	36.546111	126.775556
18	Cheongyang-gun 1	51	36.563889	126.855000
19	Cheongyang-gun 2	137	36.483056	126.773056
20	Cheongyang-gun 3	155	36.398611	126.731667
21	Cheongyang-gun 4	50	36.358611	126.856389
22	Cheongyang-gun 5	85	36.448056	126.948611
23	Taean-gun	26	36.782778	126.186111
24	Eumseong-gun	190	36.917500	127.636389
25	Jecheon-si	240	37.145556	128.132500
